# Role of ALDH2 in myocardial ischemia-reperfusion injury: using ALDH2 as a potential therapeutic target

**DOI:** 10.3389/fphar.2026.1846879

**Published:** 2026-09-03

**Authors:** Meng Sun, Zhixiong Gao, Fengyan Hu, Guo Chen

**Affiliations:** 1 Department of Anesthesiology, West China Hospital, Sichuan University, Chengdu, China; 2 Department of Anesthesiology, Institute of Anesthesiology and Critical Care Medicine, Union Hospital, Tongji Medical College, Huazhong University of Science and Technology, Wuhan, China; 3 Wuhan Britain-China School, Wuhan, China

**Keywords:** ALDH2, clinical translation, myocardial ischemia-reperfusion injury, NEtosis, target

## Abstract

Myocardial ischemia-reperfusion (I/R) injury remains a major barrier to the full benefit of timely coronary reperfusion. This review summarizes the role of mitochondrial aldehyde dehydrogenase 2 (ALDH2) as both an endogenous cardioprotective enzyme and a potential therapeutic target in myocardial I/R injury. During reperfusion, reactive oxygen species promote lipid peroxidation and the accumulation of cytotoxic aldehydes, particularly 4-hydroxy-2-nonenal (4-HNE) and malondialdehyde. ALDH2 detoxifies these aldehydes and thereby modulates several injury pathways, including mitochondrial dysfunction, neutrophil extracellular trap formation, ferroptosis, apoptosis, necroptosis, and maladaptive autophagy. Experimental studies consistently indicate that ALDH2 deficiency aggravates infarct size, inflammatory injury, aldehyde overload, and adverse ventricular remodeling, whereas ALDH2 activation or overexpression confers protection. Clinical and genetic studies further suggest that the ALDH2 rs671/ALDH2*2 loss-of-function variant may influence myocardial infarction risk, reperfusion injury severity, and the response to cardioprotective strategies, especially in East Asian populations. However, the evidence remains largely preclinical, and clinical translation will require genotype-informed patient stratification, optimization of ALDH2-targeted agonists, careful timing around reperfusion, safety evaluation, and combination strategies with established reperfusion and cardioprotective approaches.

## 1 Introduction

Cardiovascular disease is one of the leading causes of morbidity and mortality worldwide, and according to the World Health Organization, cardiovascular diseases, including coronary heart disease, account for approximately 32% of all deaths globally, a large proportion of which are due to myocardial ischemia (Timmis et al., 2020; Tsao et al., 2023; Virani et al., 2021). Timely and effective restoration of coronary blood flow to the ischemic myocardium is essential (Boag et al., 2017; Ibáñez et al., 2015). However, in the process of reperfusion to re-establish the blood supply, it can instead lead to further myocardial cell death, resulting in more severe myocardial damage. This phenomenon is known as myocardial I/R injury (Xiang et al., 2024). Prompt and effective reperfusion therapy remains the most effective clinical tool for treating myocardial ischemia. A variety of therapeutic strategies have emerged to reduce the damage caused by reperfusion, including physical therapy and pharmacological therapy (Zhang et al., 2024; Li et al., 2023). However, the clinical outcomes of these treatments are not satisfactory, and there is a lack of effective treatments to mitigate myocardial I/R injury. Therefore, targeting to reduce the harm of reperfusion injury has become one of the hotspots in the field of cardiovascular disease research.

Aldehyde dehydrogenase 2 (ALDH2) not only detoxifies the products of alcohol metabolism but also contributes to the removal of endogenous aldehydes produced during myocardial I/R injury, including 4-hydroxy-2-nonenal (4-HNE) and malondialdehyde (MDA) (Bai et al., 2023; Liu et al., 2023). 4-HNE can be metabolized by ALDH2 to the less reactive 4-hydroxy-2-nonenoic acid, thereby mitigating aldehyde-mediated mitochondrial and contractile injury (Budas et al., 2009). In this review, we summarize the cardioprotective mechanisms mediated by ALDH2 in myocardial I/R injury, distinguish the relative strength of evidence across mechanistic pathways, and discuss the translational barriers that must be addressed before ALDH2-targeted therapies can be tested rigorously in clinical reperfusion settings.

## 2 Myocardial I/R injury and aldehyde accumulation

Myocardial I/R injury is most commonly associated with ischemic heart disease, during which myocardial cells do not receive enough oxygen, resulting in severe tissue hypoxia (Thygesen et al., 2018). With the use of techniques such as coronary angioplasty, coronary artery bypass grafting and other techniques to recanalise the blood vessels and restore the blood supply to the ischemic tissues, the sudden reoxygenation generates a large amount of reactive oxygen species (ROS), which disrupts intracellular calcium homeostasis and further increases tissue damage (Xiang et al., 2024; Cadenas, 2018). To date, readmission rates for injury due to reperfusion therapy remain high in the United States, and there is an urgent need to ameliorate the problem of possible injury from reperfusion. Unfortunately, there is still a lack of reliable therapeutic modalities that can be used for clinical translation (Heusch, 2020).

A major cause of worsening ischemic injury due to reperfusion is free radical-induced lipid peroxidation with cellular destruction (Zweier et al., 2006). During ischemia, hypoxia leads to anaerobic metabolism, resulting in the accumulation of metabolic by-products such as lactate and ATP depletion (Stanley et al., 2005). The sudden influx of oxygen during reperfusion generates large amounts of ROS leading to oxidative stress. Under oxidative stress, reactive oxygen species attack unsaturated fatty acids in the cell membrane, undergo lipid peroxidation, and produce a variety of reactive aldehydes, further exacerbating oxidative damage (Ayala et al., 2014). Among the several reactive aldehydes produced, HNE and MDA are the most potent and toxic substances that promote ischemic injury (Uchida, 2003). Unlike the site-specific damage caused by free radicals, aldehydes can diffuse away from the site of their production, thereby prolonging and amplifying the damage.

Aldehydes can alter the proteasome and reduce its peptidase activity (Okada et al., 1999). After myocardial I/R, reactive aldehydes can directly inhibit mitochondrial respiration and also reduce the activity of myocardial enzymes involved in glycolysis (Lucas and Szweda, 1999). In addition, excess aldehydes disrupt the interaction between actin and myosin, thereby damaging cardiomyocytes and impairing cardiac function (Budas et al., 2009).

## 3 ALDH2 and aldehyde metabolism

ALDH2 is a tetrameric variable mitochondrial enzyme located in the mitochondrial matrix (Dabravolski and Isayenkov, 2021). It also has dehydrogenase, reductase and esterase activities (Black et al., 2009). It is the main enzyme involved in detoxification in the body (Vasiliou and Nebert, 2005). ALDH2 plays a key role in ethanol metabolism, catalysing the oxidation of acetaldehyde, the major metabolite of ethanol, to acetic acid (Crabb et al., 2004). Acetaldehyde is highly toxic and carcinogenic, and needs to be rapidly metabolised; the presence of ALDH2 significantly reduces the accumulation of acetaldehyde in the body, mitigating its toxicity and carcinogenic risk (Seitz and Stickel, 2007). The oxidised acetic acid can further enter the tricarboxylic acid cycle and participate in energy metabolism to provide energy to the cells (Edenberg, 2007).

**FIGURE 1:**
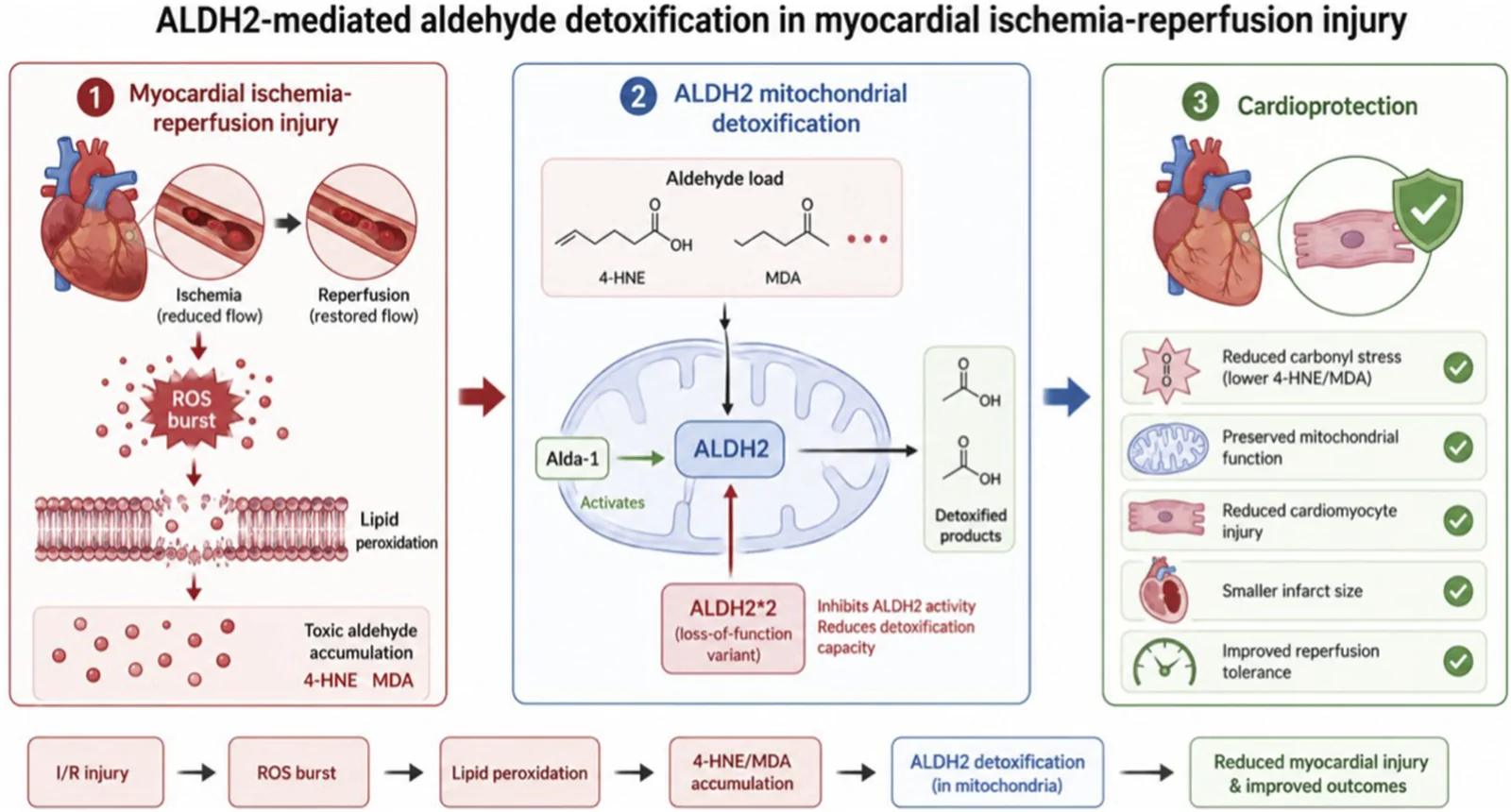
Focused schematic of ALDH2-mediated aldehyde detoxification during myocardial ischemia-reperfusion injury.

ALDH2 not only detoxifies the products of alcohol metabolism, but also contributes to the removal of other endogenous aldehydes produced during myocardial I/R injury, including 4-HNE and MDA (Chen et al., 2008). 4-HNE can be metabolised by ALDH2 to the non-reactive 4-HNA, thereby mitigating the cardiac damage caused by toxic aldehydes (Budas et al., 2009). This aldehyde-detoxification axis is summarized schematically in Figure 1.

It has been shown that point mutations in active ALDH2*1 lead to ALDH2 defects, producing ALDH2*2 that is not enzymatically active and unable to degrade aldehydes in the body (Yoshida et al., 1984). Polymorphisms in ALDH2 lead to elevated levels of acetaldehyde in the body. After heavy drinking, the level of acetaldehyde in the blood of people with impaired ALDH2 function increases about 10-fold compared to people with normal ALDH2.

Beyond genetic polymorphisms, ALDH2 activity is also subject to post-translational regulation. A recent study published in Circulation has identified the E3 ubiquitin ligase Hrd1 as a novel upstream regulator of ALDH2 in myocardial I/R injury (Shuolin et al., 2026). Mechanistically, Hrd1 promotes K33-linked polyubiquitination of ALDH2, which inhibits the formation of its active tetrameric conformation, thereby suppressing ALDH2 enzymatic activity and exacerbating endothelial dysfunction through the NO/cGMP/PKG signaling pathway (Shuolin et al., 2026). Genetic ablation or pharmacological inhibition of Hrd1 significantly alleviated myocardial infarction and endothelial dysfunction in I/R models, suggesting that targeting Hrd1 represents an alternative strategy to enhance ALDH2 activity and confer cardioprotection (Shuolin et al., 2026). These findings add a new layer of complexity to the regulation of ALDH2 activity and open new therapeutic avenues for myocardial I/R injury.

## 4 Experimental evidence for protection against myocardial I/R injury by ALDH2

The protective role of ALDH2 in myocardial I/R injury has also been validated in multiple animal studies (Chen et al., 2008). Previous research has shown that immune responses mediated by innate immune cells such as macrophages and neutrophils play a central role in myocardial I/R injury (Wang et al., 2022). Activated neutrophils can release neutrophil extracellular traps (NETs), leading to microvascular obstruction. This is a major factor contributing to I/R injury and infarct size (Bonaventura et al., 2018). Limiting neutrophil infiltration can salvage myocardial tissue. In light of this, Yang et al. assessed whether ALDH2 could alleviate myocardial I/R injury by influencing the formation of neutrophil extracellular traps. They used ALDH2-KO mice and, by comparing with WT mice, found that the infarct area (IA) to area at risk (AAR) ratio and cTnT levels were higher in KO mice. Flow cytometry data showed a significant increase in the infiltration of cardiomyocytes and macrophages in the hearts of KO mice. The enhancement of NETosis was reflected in a significant increase in citH3 levels in the AAR tissue of KO mice. After treatment with NETosis-targeting drugs, the degree of myocardial injury in ALDH2-KO mice was significantly improved. This study confirms that the absence of ALDH2 exacerbates myocardial I/R injury, and NETosis plays a critical role in this process (Yang et al., 2024). Ma et al. confirmed in ALDH2-KO mice that the absence of ALDH2 significantly exacerbates myocardial infarct size and worsens left ventricular systolic dysfunction. However, after overexpressing ALDH2, they eliminated the increase in 4-HNE caused by ischemia and reperfusion, reversed its induced cardiomyocyte dysfunction, and promoted recovery of left ventricular function in mice, demonstrating a cardioprotective effect (Ma et al., 2011).

In order to reduce the risks associated with reperfusion, various pharmacological and mechanical therapeutic measures have been developed (Heusch, 2015). These include remote ischemic preconditioning and remote ischemic postconditioning (Heusch, 2015). Due to the unpredictability of ischemic events, the clinical application of remote ischemic preconditioning is limited (Hausenloy and Yellon, 2008). Unlike remote ischemic preconditioning, remote ischemic postconditioning is performed after ischemia and is more suitable for clinical use. Its clinical significance in cardiac protection was confirmed in 2013, although the underlying molecular mechanisms remain unclear (Verouhis et al., 2016). Zhang et al. explored this issue and found that the mRNA expression of ALDH2 was significantly upregulated in mice subjected to myocardial I/R injury after remote ischemic postconditioning. Silencing ALDH2 eliminated the protective effect of remote ischemic postconditioning, as evidenced by a significant decrease in cell viability and an increase in apoptosis-related proteins in H9C2 cells. These findings strongly confirm that sufficient ALDH2 activity is an important safeguard for reducing myocardial injury following I/R (Zhang et al., 2018).

To improve readability and to separate the relative evidentiary support for each mechanism, the main cited studies are summarized in Table 1. The table distinguishes direct preclinical evidence, mechanistic pathway evidence, and early clinical/genetic evidence, because these categories should not be interpreted as having equivalent translational certainty.

**TABLE 1:** Summary of major cited evidence on ALDH2 in myocardial ischemia-reperfusion injury.

Mechanistic domain	Representative study	Model or evidence type	Intervention or approach	Key finding	Evidence strength
Aldehyde detoxification	Chen et al. (2008)	Rodent cardiac I/R and biochemical assays	Alda-1 activation of ALDH2	ALDH2 activation reduced ischemic cardiac damage and limited 4-HNE-associated injury	Strong preclinical; indirect clinical relevance
Autophagy paradox	Yang et al. (2024)	ALDH2 overexpression/deficiency in I/R models	Genetic ALDH2 modulation	ALDH2 promoted adaptive autophagy during ischemia but restrained excessive autophagy during reperfusion	Moderate mechanistic evidence
NETosis and inflammation	Bonaventura et al. (2018)	ALDH2-knockout mice and STEMI nested case-control cohort	NETosis inhibition and LTC4 pathway analysis	ALDH2 deficiency enhanced ER stress, Mgst2-LTC4 signaling, NETosis, and adverse remodeling markers	Strong mechanistic; emerging clinical biomarker evidence
Ferroptosis	Liu et al. (2023)	Myocardial I/R and cardiomyocyte injury models	Alda-1, iron chelation, and 4-HNE-GPX4 analysis	4-HNE promoted GPX4 dysfunction and ferroptosis; ALDH2 activation attenuated lipid peroxidation injury	Moderate mechanistic evidence
Mitochondrial protection	Wang et al.(2017); Gu et al. (2013)	H/R cardiomyocytes and post-MI remodeling models	ALDH2 activation or expression modulation	ALDH2 preserved mitochondrial morphology, respiratory reserve, and downstream survival signaling	Moderate preclinical evidence
Clinical polymorphism	Chen et al. (2014); Takagi et al. (2002)	STEMI and acute myocardial infarction cohorts	ALDH2 rs671 genotype analysis	ALDH2*2 was associated with greater injury burden or AMI risk in East Asian populations	Emerging clinical association; requires prospective validation
CABG translation	Li et al. (2024)	Off-pump CABG cohort	ALDH2 genotype and aldehyde-adduct assessment	ALDH2*2 carriers showed more postoperative aldehyde adducts, higher cTnI, and longer ICU stay	Clinical association; intervention untested

The evidence-strength labels in Table 1 were assigned qualitatively according to evidence type and translational certainty: direct genetic or pharmacological intervention in animal I/R models was considered strong preclinical evidence; direct pathway perturbation with mechanistic endpoints was considered strong or moderate mechanistic evidence depending on reproducibility and clinical support; cell/animal data without human validation were considered moderate preclinical evidence; and genotype, biomarker, or perioperative cohort findings without prospective intervention were classified as emerging clinical or clinical association evidence. This classification was intended for narrative synthesis and should not be interpreted as a formal GRADE assessment.

## 5 Protective mechanisms of ALDH2 against myocardial I/R injury

The cardioprotective role of ALDH2 should be viewed as a network effect rather than a single linear pathway. Aldehyde detoxification is the biochemical core, but its consequences extend to inflammatory activation, mitochondrial stability, regulated cell death, and remodeling. The sections below therefore discuss each mechanism separately while noting areas where the evidence remains incomplete or primarily model-based.

### 5.1 ALDH2 and NETosis

**FIGURE 2:**
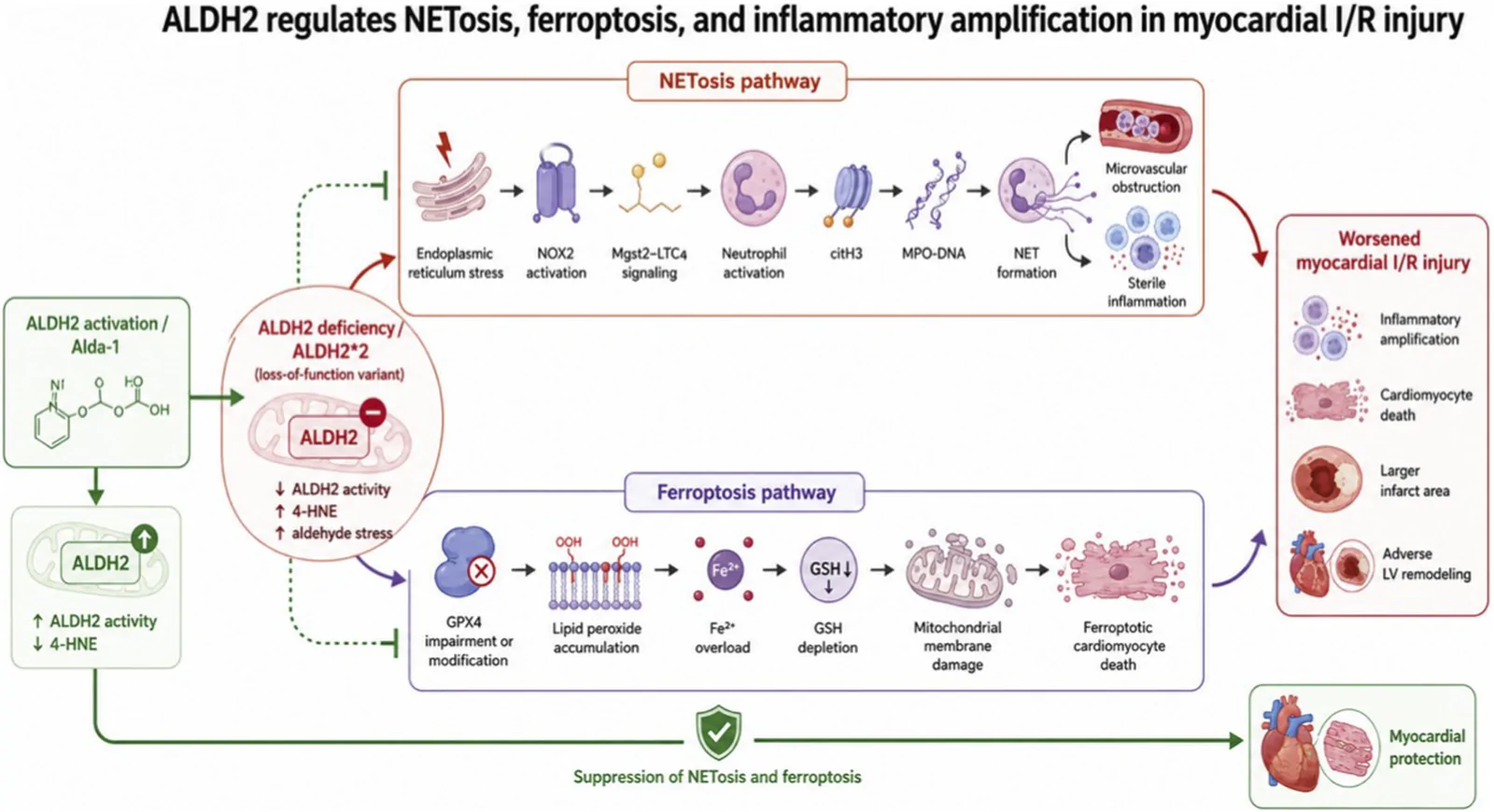
Focused schematic of ALDH2-related regulation of NETosis, ferroptosis, and inflammatory amplification in myocardial I/R injury.

Inflammatory response is one of the important mechanisms contributing to myocardial I/R injury (Frang and ogiannis, 2012). Neutrophils play a critical role in the inflammatory response (Vinten-Johansen, 2004). However, excessive accumulation and activation of neutrophils can lead to the massive release of damage-associated molecular patterns and inflammatory factors, resulting in a series of severe injuries to the myocardium (Lindsey et al., 2001). Additionally, activated neutrophils release NETs, which serve as a hub linking sterile inflammation and thrombosis. NETs can also promote the formation of reactive oxygen species and the secretion of inflammatory factors, further amplifying the inflammatory response (Papayannopoulos, 2018). Yang and colleagues found that ALDH2-KO mice exhibited more severe myocardial I/R injury along with increased NETosis, as evidenced by significantly elevated levels of citH3 in the AAR tissue (Yang et al., 2024). To further investigate the mechanism of NETosis induced by ALDH2 deficiency, Yang et al. examined markers of lipid peroxidation and endoplasmic reticulum stress (ERS) in neutrophils from mice. They found an accumulation of both types of substances in ALDH2-KO neutrophils. Subsequently, the team used N-acetylcysteine to clear lipid peroxides or inhibit ERS, and observed significant reductions in NOX2 and citH3 levels. This indicates that NOX2-dependent NETosis induced by ALDH2 deficiency may be mediated by ERS (Yang et al., 2024). To identify downstream target genes, the authors performed proteomic analysis and selected Mgst2 as a downstream gene. Mgst2 is an isoenzyme of LTC4, which catalyzes the production of LTC4 through conjugation with glutathione. The Mgst2-LTC4 pathway is crucial for endoplasmic reticulum stress signaling (Zhao et al., 2021). To determine whether this pathway plays a role in ALDH2-mediated NETosis, siRNA (si-Mgst2) and the LTC4 antagonist Prucalopride were used. As expected, inhibition of this signaling pathway prevented the exacerbation of NETosis induced by ALDH2 deficiency and alleviated ERS (Yang et al., 2024). The integrated regulation of NETosis, ferroptosis, mitochondrial dysfunction, and inflammatory amplification is illustrated in Figure 2.

### 5.2 ALDH2 and ferroptosis

Ferroptosis is a novel form of iron-dependent programmed cell death that is distinct from apoptosis, necrosis, and autophagy (Dixon et al., 2012). Its role in myocardial I/R injury has been confirmed by numerous studies (Fang et al., 2019). Recent evidence has highlighted a close functional interplay between ALDH2 and ferroptosis in the pathogenesis of myocardial I/R injury (Han and Zhai, 2026). During the process of myocardial ischemia and reperfusion, iron metabolism becomes disrupted; excess iron can undergo Fenton reactions with H2O2 to generate large amounts of hydroxyl radicals, disrupting redox homeostasis, triggering lipid peroxidation, and ultimately leading to cell death (Toyokuni, 2009).

4-HNE is an endogenous toxic aldehyde produced during myocardial I/R (Esterbauer et al., 1991). It can inhibit GPX4 expression by promoting the ubiquitination of GPX4 on K48 chains, leading to its ubiquitin-proteasome degradation. GPX4 is a GSH-dependent enzyme that can eliminate excess peroxides and hydroxyl radicals, making it a key regulatory protein in inhibiting ferroptosis (Yang et al., 2014).

In ALDH2-deficient mice, the accumulation of 4-HNE and ferroptosis induced by myocardial I/R are significantly exacerbated, as indicated by changes in iron accumulation, MDA production, and GSH depletion—markers related to ferroptosis. Activation of ALDH2 can alleviate the accumulation of 4-HNE and ferroptosis (Chen et al., 2008). In mice treated with the iron chelator DXZ or the ALDH2 agonist Alda-1, 4-HNE levels significantly decreased, and the area of myocardial infarction and CK-MB levels also notably reduced. Additionally, the increase in Fe2+, decrease in GSH, and expression of related proteins were restored (Liu et al., 2023).

At the cellular level, it was further confirmed that the cytotoxicity of 4-HNE is associated with dysfunction of GPX4 (Uchida, 2003). In H9C2 cells, treatment with 4-HNE resulted in a significant increase in Fe2+ and a reduction in the expression and activity of GPX4 protein (Liu et al., 2023). Moreover, mitochondrial vacuolization and membrane rupture were observed. Conversely, DXZ and Alda-1 were able to reverse these changes. Immunofluorescence analysis showed increased binding of 4-HNE to GPX4 in ALDH2-KO mice. 4-HNE can react with the cysteine residues of GPX4 through Michael addition, leading to covalent modification and carbonylation of the protein, thereby inhibiting its function. The construction of carbonylation-defective mutants demonstrated that carbonylation is crucial for 4-HNE-induced degradation of GPX4 (Liu et al., 2023).

### 5.3 ALDH2 and mitochondrial dysfunction

Mitochondria are central to energy metabolism in cardiac myocytes, and mitochondrial dysfunction is a key factor in myocardial I/R injury (Murphy and Steenbergen, 2008). ALDH2 protects mitochondrial structure and function through multiple mechanisms (Ma et al., 2011).

Firstly, ALDH2 is essential for maintaining mitochondrial respiratory function. Studies have shown that treatment of H9C2 cardiomyocytes with the ALDH2 inhibitors disulfiram or 4-HNE significantly reduces mitochondrial oxygen consumption rate (OCR) and mitochondrial respiratory reserve capacity, thereby triggering an energy metabolic crisis unrelated to cell death (Mali et al., 2016). Impaired mitochondrial respiratory function has also been observed in cardiomyocytes from ALDH2-knockout (ALDH2^−/−^) mice, further confirming the importance of endogenous ALDH2 in maintaining baseline mitochondrial function (Wang et al., 2017; Gu et al., 2013).

Secondly, ALDH2 regulates mitochondrial dynamics, particularly the balance between mitochondrial fission and fusion. In H9C2 cells subjected to hypoxia/reoxygenation (H/R) injury, the phosphorylation level of the mitochondrial fission protein Drp1 at serine 616 is significantly elevated, leading to mitochondrial fragmentation and subsequent apoptosis. Overexpression or activation of ALDH2 significantly reversed Drp1 phosphorylation, inhibited excessive mitochondrial fission, and thereby protected mitochondrial morphology (Zhang et al., 2020). This process may be mediated by inhibiting the phosphorylation of AMPK, an upstream regulator of Drp1 (Zhang et al., 2020).

Furthermore, ALDH2 maintains the mitochondrial membrane potential (ΔΨm) by inhibiting the opening of the mitochondrial permeabilisation transition pore (mPTP). Under I/R stress, mitochondria in the hearts of ALDH2^−/−^ mice exhibited more severe ΔΨm depolarisation. Treatment with a GSK-3β inhibitor or PKC-δ shRNA ameliorates this phenomenon, suggesting that ALDH2 may suppress mPTP opening and prevent the release of pro-apoptotic factors (such as cytochrome c) by regulating PKC isoforms (promoting PKC-ε and inhibiting PKC-δ) and downstream GSK-3β signaling (Wang et al., 2017; Gu et al., 2013). ALDH2 also attenuates the downregulation of mitochondrial electron transport chain complexes I and V induced by I/R, thereby maintaining the efficiency of oxidative phosphorylation (Gomes et al., 2015; Zhang et al., 2012).

### 5.4 ALDH2 and aldehyde toxicity (4-HNE)

The most direct protective effect of ALDH2 stems from its enzymatic activity—namely, the metabolism and clearance of toxic aldehydes generated during I/R, primarily 4-HNE and MDA. Under physiological conditions, low concentrations of 4-HNE act as signaling molecules; however, under I/R-induced oxidative stress, its concentration rises sharply, leading to ‘aldehyde overload’ (Zhang et al., 2012).

Elevated 4-HNE forms adducts with proteins, lipids and DNA, leading to loss of protein function, inhibition of enzyme activity and damage to cellular structures. For example, 4-HNE can directly modify and inhibit the activity of complexes in the mitochondrial electron transport chain (Mali et al., 2016; Gomes et al., 2015). More critically, 4-HNE itself inhibits ALDH2 activity, thereby creating a vicious cycle. Reduced ALDH2 activity diminishes the clearance of 4-HNE, while accumulated 4-HNE further inactivates ALDH2 (Chen et al., 2008). The ALDH2 agonist Alda-1 not only directly activates ALDH2 but also acts as a “chemical chaperone”, protecting ALDH2 from 4-HNE-induced inactivation and thereby breaking this cycle (Lagranha et al., 2010).

Excessive aldehyde toxicity also manifests through the modification of key signaling proteins. Studies have found that 4-HNE can bind directly to receptor-interacting protein kinase 1 (RIP1), inhibiting its K48-linked ubiquitination and subsequent proteasomal degradation, thereby stabilising the RIP1 protein and activating necrotic apoptosis in cardiomyocytes (Zhai et al., 2021). This reveals a mechanism whereby 4-HNE-mediated aldehyde toxicity exerts its effects via a programmed necrosis pathway distinct from apoptosis. In ALDH2 transgenic (ALDH2-Tg) mice, 4-HNE levels in the myocardium were significantly reduced following I/R injury, while the formation of the RIP1-RIP3 necrotic complex was reduced, suggesting that enhanced 4-HNE clearance can effectively inhibit this necrotic apoptosis pathway (Zhai et al., 2021). While ALDH2 clusters as a multi-pathway Regulated Cell Death (RCD) inhibitor, a critical unresolved question is the spatiotemporal hierarchy of these death modes during hyperacute reperfusion. For instance, whether 4-HNE-induced GPX4 carbonylation (ferroptosis) precedes or runs parallel to RIP1/RIP3 complex stabilization (necroptosis) remains obscured. Moreover, most preclinical models utilize prolonged ischemia (>30 min), which tips the balance toward necrosis. In clinical post-PCI settings with shorter ischemic windows, the relative contribution of ALDH2 in mitigating apoptosis versus ferroptosis requires further genetic dissection using cell-type-specific inducible knockout models. Furthermore, aldehyde toxicity can induce endoplasmic reticulum stress, thereby activating NOX2-dependent NET formation and exacerbating I/R injury (Pan et al., 2021).

While ALDH2 clusters as a multi-pathway Regulated Cell Death (RCD) inhibitor, a critical unresolved question is the spatiotemporal hierarchy of these death modes during hyperacute reperfusion. For instance, whether 4-HNE-induced GPX4 carbonylation (ferroptosis) precedes or runs parallel to RIP1/RIP3 complex stabilization (necroptosis) during the immediate minutes of blood flow restoration remains obscured. Moreover, most preclinical models utilize prolonged ischemia (>30 min), which tips the balance toward necrosis. In clinical post-PCI settings with shorter ischemic windows, the relative contribution of ALDH2 in mitigating apoptosis versus ferroptosis requires further genetic dissection using cell-type-specific inducible knockout models.

### 5.5 ALDH2 and autophagy

Autophagy plays a ‘double-edged sword’ role in myocardial I/R injury (Matsui et al., 2007). During ischemia, moderate autophagy exerts a protective effect by clearing damaged organelles and recycling nutrients; however, during reperfusion, excessive activation of autophagy may promote cell death (Ma et al., 2012). It is precisely through the precise regulation of this process that ALDH2 exerts its cardioprotective effects.

Ma et al. found that in a systemic I/R model, ALDH2 regulates autophagy via two distinct mechanisms (Gu et al., 2013). During the ischemic phase, ALDH2 activates AMPK and inhibits mTOR, thereby inducing autophagy, which aids cell survival under hypoxic conditions. During the reperfusion phase, ALDH2 conversely activates Akt, inhibiting excessive autophagy via the Akt-mTOR pathway, thereby preventing cell death due to autophagy. This precise regulation of the ‘autophagy paradox’—namely, the promotion of autophagy during ischemia and its inhibition during reperfusion—relies on the spatiotemporal specificity of ALDH2’s regulation of the AMPK and Akt signaling pathways (Gu et al., 2013; Zhang and Ren, 2010).

Conversely, when ALDH2 function is lost (e.g., through the use of inhibitors or the presence of the ALDH2*2 mutation), this fine-tuned regulation is disrupted. During ischemia, impaired AMPK signaling leads to insufficient autophagy initiation, resulting in the accumulation of damaged proteins and organelles (such as dysfunctional mitochondria), which in turn exacerbates cellular damage (Gu et al., 2013). During reperfusion, insufficient Akt signaling leads to excessive mTOR inhibition, abnormally increasing autophagic flux and inducing autophagic cell death. Thus, by maintaining the dynamic balance of autophagy, ALDH2 ‘harnesses the benefits while avoiding the drawbacks’ at different stages of I/R, which constitutes one of the key mechanisms underlying its cardioprotective effects.

However, the “autophagy paradox” managed by ALDH2 introduces a substantial translational dilemma. Although endogenous ALDH2 orchestrates a delicate homeostatic balance—bifurcating its downstream signaling to switch from AMPK-mediated autophagy activation during ischemia to Akt-mTOR-mediated inhibition during reperfusion—pharmacological interventions lack this inherent spatiotemporal feedback mechanism. Currently, most supporting evidence relies heavily on steady-state Western blot assessments. These snapshot measurements reflect net protein accumulation (such as LC3-II or p62 levels) at single time points rather than the true kinetic dynamics of the pathway. Consequently, a static increase in autophagic markers can easily be misinterpreted: it remains unclear whether ALDH2 activation genuinely drives adaptive autophagic clearance or inadvertently creates a metabolic bottleneck by impairing downstream lysosomal degradation in the context of various comorbidities. To resolve these conflicting mechanistic interpretations, future research priorities must shift from steady-state protein snapshots to dynamic *in vivo* autophagic flux assays—using tandem fluorescent reporters or real-time intravital imaging—to precisely track how ALDH2-targeted agonists modulate the continuous lifespan of autophagosomes during hyperacute reperfusion.

### 5.6 ALDH2 and apoptosis

Apoptosis is the primary form of cardiomyocyte death following I/R. ALDH2 exerts its anti-apoptotic effects through multiple pathways. At the whole-animal level, compared with wild-type mice, ALDH2^−/−^ mice exhibit a larger area of myocardial infarction and a higher myocardial cell apoptosis index following I/R, accompanied by a significant increase in the levels of cleaved caspase-3 and cleaved caspase-9 (Wang et al., 2017; Gu et al., 2013). This directly demonstrates the necessity of ALDH2 in inhibiting apoptosis following I/R.

At the cellular level, ALDH2 clears signaling molecules that activate pro-apoptotic pathways such as JNK/p53 by metabolising toxic aldehydes such as 4-HNE (Gomes et al., 2015). Research by Gu et al. indicates that 4-HNE-induced carbonyl stress can directly lead to SIRT1 inactivation, thereby increasing the sensitivity of cardiomyocytes to I/R injury, while activation of ALDH2 can reverse this process (Gu et al., 2013). Furthermore, ALDH2 can inhibit the opening of the mitochondrial permeabilisation transition pore (mPTP) by maintaining mitochondrial function, thereby reducing the release of cytochrome c from the mitochondria into the cytoplasm and blocking the activation of the downstream caspase cascade (Wang et al., 2017). ALDH2 can also regulate Bcl-2 family proteins to inhibit apoptosis. In the remote ischemia post-conditioning (RIPostC) model, upregulation of ALDH2 was accompanied by a significant increase in the Bcl-2/Bax ratio in myocardial tissue, suggesting that ALDH2 helps maintain the balance between the anti-apoptotic protein Bcl-2 and the pro-apoptotic protein Bax, thereby inhibiting the mitochondrial-mediated intrinsic apoptotic pathway (Yu et al., 2014). Similarly, in H9C2 cells subjected to hypoxia/reoxygenation (H/R) injury, the cardioprotective effect of baicalin depends on ALDH2-mediated downregulation of Bax, upregulation of Bcl-2, and inhibition of cytochrome c release (Jiang et al., 2018).

## 6 Clinical significance and translational roadmap

A substantial amount of preclinical evidence confirms the contribution of ALDH2 to myocardial protection following I/R (Chen et al., 2008). This offers a new and promising therapeutic target for mitigating damage from reperfusion in clinical settings (Chen et al., 2014). Yang et al. further validated their findings in clinical patients based on animal experiments (Yang et al., 2024). They conducted a nested case-control study involving STEMI patients who underwent PCI treatment, suggesting that patients with the ALDH2 rs671 mutant genotypes (GA, AA) had higher levels of MPO-DNA and LTC4 compared to those with the wild-type genotype (GG). Patients with adverse left ventricular remodeling outcomes typically exhibited higher levels of MPO-DNA and LTC4. Subsequent ROC analysis indicated that incorporating MPO-DNA and LTC4 into traditional myocardial infarction risk models significantly improved the predictive capacity for left ventricular remodeling (LVR), with AUC values of 0.721 and 0.701, respectively (Yang et al., 2024).

It has been reported that the ALDH2*2 genotype is a risk factor for AMI in East Asians (Takagi et al., 2002). In Japanese patients with acute ST-segment elevation myocardial infarction, the frequency of the ALDH2*2 allele was 37.7%, which is significantly higher than the 24.3% found in the general Japanese population (Ishida et al., 2022). Furthermore, ALDH2*2 carriers tend to have higher plasma levels of LDH, cTnT, and BNP, as well as poorer left ventricular ejection fraction (LVEF). The functional deficiency of ALDH2 exacerbates the severity of I/R injury. Subsequent studies in Korean and Han Chinese populations further supported this finding (Ishida et al., 2022; Li et al., 2024).

While remote ischemic preconditioning presents a viable method to reduce I/R injury, Contractor et al. discovered in humans that individuals with ALDH2 polymorphisms showed reduced forearm blood flow responsiveness to acetylcholine before I/R, failing to induce protective effects on endothelial cells. In contrast, individuals with functional ALDH2 exhibited effective protective responses to remote ischemic preconditioning (Contractor et al., 2013).

**FIGURE 3:**
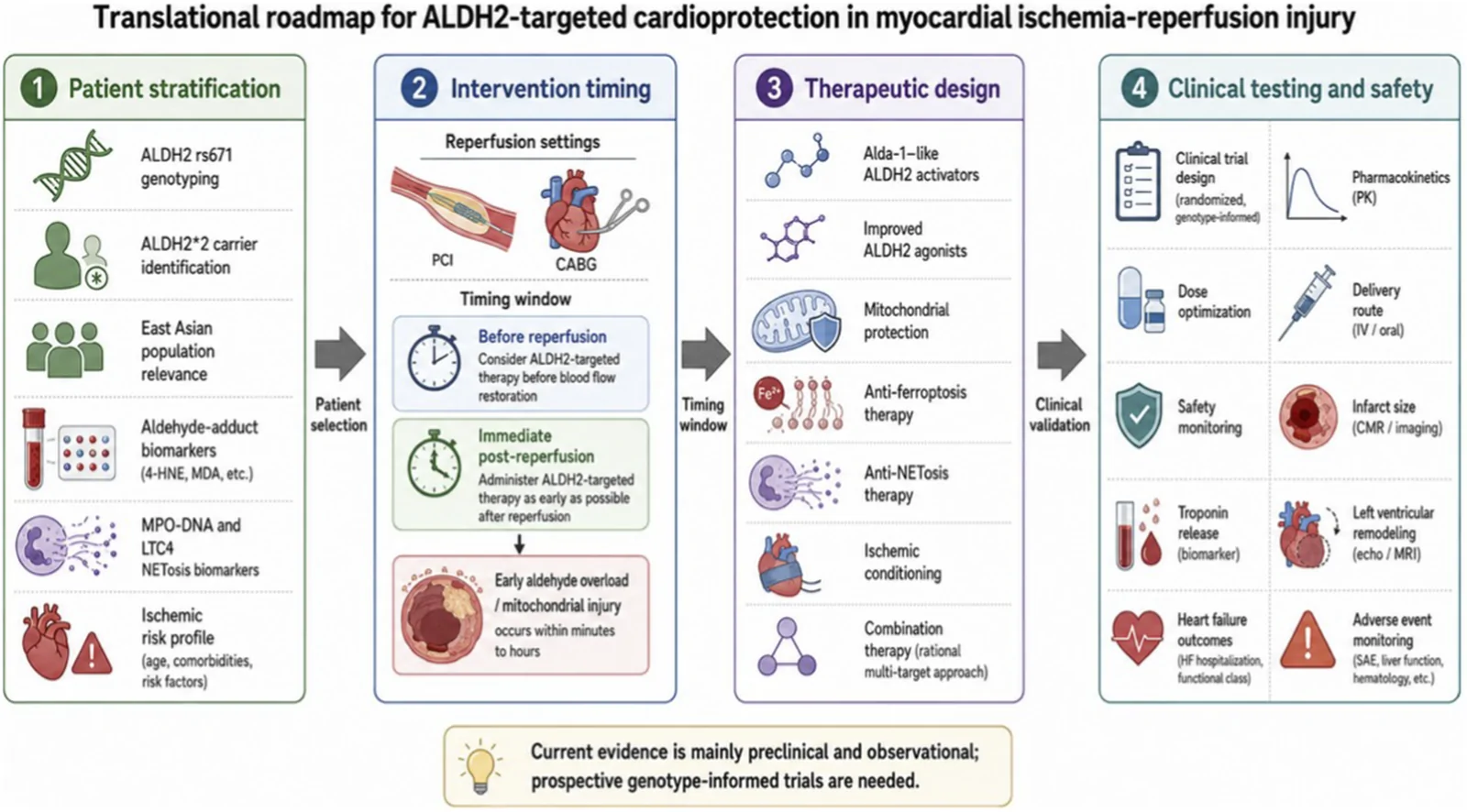
Proposed translational roadmap for ALDH2-targeted cardioprotection, including patient selection, intervention timing, therapeutic design, and clinical testing.

To investigate the impact of the ALDH2*2 genotype on cardiac protection after CABG surgery, Zhang et al. collected preoperative and postoperative plasma samples from 207 patients undergoing selective off-pump CABG. The analysis revealed significantly increased levels of circulating HNE and MDA adducts in samples with ALDH2 functional deficiencies after surgery, along with markedly elevated postoperative myocardial injury markers such as cTnI (Gong et al., 2019). Additionally, ALDH2*2 carriers spent longer time in the intensive care unit. These findings collectively suggest that targeting ALDH2 holds promise as a new strategy for treating myocardial I/R injury. The proposed genotype-informed translational pathway and key implementation barriers are summarized in Figure 3; Table 2.

**TABLE 2:** Therapeutic roadmap and translational barriers for ALDH2-targeted cardioprotection.

Roadmap element	Current basis	Main barrier	Proposed next step
Patient stratification	ALDH2*2 is linked to worse AMI/I/R phenotypes (Chen et al., 2014; Takagi et al., 2002)	Genotype is rarely used in reperfusion trials	Genotype-stratified cohorts and prespecified subgroup analyses
ALDH2 agonist development	Alda-1 activates ALDH2 and stabilizes ALDH2*2 [27; 68]	Dose, delivery, and human safety remain uncertain	Optimize compounds and perform early pharmacokinetic/safety studies
Timing of intervention	Aldehyde and mitochondrial injury begin early (Heusch, 2020; Chen et al., 2008)	Emergency PCI leaves little pretreatment time	Define ambulance, catheterization laboratory, CABG, and postconditioning windows
Combination strategies	ALDH2 intersects with ferroptosis, NETosis, mitochondria, and conditioning (Liu et al., 2023; Bonaventura et al., 2018; Verouhis et al., 2016)	Single-pathway therapy may be insufficient	Test biomarker-guided combinations with complementary cardioprotection
Outcome and safety	ALDH2*2 relates to biomarkers, LVR, and postoperative injury (Bonaventura et al., 2018; Li et al., 2024)	Intervention and target-engagement data are limited	Track infarct size, troponin, LVR, aldehyde adducts, MPO-DNA/LTC4, and adverse events

Several translational issues deserve emphasis. First, ALDH2-targeted therapy is unlikely to be a one-size-fits-all strategy; genotyping for ALDH2 rs671, together with aldehyde-adduct or NETosis-related biomarkers, may help identify patients most likely to benefit (Yang et al., 2024; Ishida et al., 2022; Li et al., 2024; Gong et al., 2019). Second, the therapeutic window is narrow. Because aldehyde overload, mitochondrial permeability transition, NETosis, and ferroptosis occur early during reperfusion, ALDH2 agonists may need to be administered before or immediately after reperfusion in PCI, CABG, or predictable high-risk ischemic settings (Heusch, 2020; Chen et al., 2008; Gong et al., 2019). Furthermore, future preclinical evaluations should deliberately test varying durations of ischemia. Most current preclinical studies employ standard 30–60 min ischemic periods, which may not fully recapitulate the complex clinical reality where patients often experience prolonged delays before coronary flow restoration. Given that ALDH2 substrates (e.g., 4-HNE) accumulate during ischemia itself, it is critical to assess whether ALDH2 activation remains protective after extended ischemic times. If mitochondrial damage or downstream death signals have already become irreversible after prolonged ischemia, ALDH2 activation alone may not confer substantial benefit. Thus, defining the therapeutic time window for ALDH2-targeted therapy is essential to rationally design clinical trials and avoid testing the strategy in ineffective scenarios. Third, Alda-1 remains primarily an experimental tool compound rather than an established clinical drug. Structural and kinetic studies show that Alda-1 can act as both an ALDH2 agonist and a chemical chaperone for the ALDH2*2 variant, but its concentration-dependent behavior, delivery route, pharmacokinetics, and long-term safety require further optimization before clinical translation (Perez-Miller et al., 2010; Belmont-Díaz et al., 2016).

Encouragingly, recent medicinal chemistry efforts have led to the development of novel triazole-based ALDH2 activators with significantly improved water solubility and activation potency. Representative compound Z17 achieved an ALDH2 activation fold of 5.4, representing 304% of the activity of Alda-1, which is the highest activity reported to date to our knowledge (Zhao et al., 2025). In a mouse model of myocardial I/R injury, intraperitoneal administration of Z17 significantly improved cardiac function (ejection fraction by 41% and fractional shortening by 36%) and reduced myocardial necrosis (infarct size by 38%, LDH by 35%, and CK-MB by 69%) (Zhao et al., 2025). These findings address key limitations of Alda-1 and provide a strong foundation for further development of ALDH2 activators as viable therapeutic agents for myocardial I/R injury.

Combination therapy is another important direction. ALDH2 activation may be most useful when paired with reperfusion strategies, ischemic conditioning, anti-ferroptotic interventions, NETosis inhibition, or mitochondrial protective agents. Such combinations should be tested with prespecified endpoints that include infarct size, cardiac troponin release, left ventricular remodeling, heart failure hospitalization, and safety signals. Future trials should also avoid relying solely on broad cardiovascular outcomes and should include mechanistic biomarkers that can confirm target engagement.

Beyond compound optimization, another critical limitation in current mechanistic frameworks is the heavy reliance on healthy, young rodent models. Clinical myocardial I/R rarely occurs in a metabolic vacuum. Standard comorbidities, such as Type 2 Diabetes and aging, are notorious for inducing baseline mitochondrial hyperacetylation and oxidative stress, which may independently suppress ALDH2 activity regardless of the rs671 genotype. Conflicting reports exist regarding whether ALDH2 upregulation can fully override the severe downstream transcriptomic rewiring induced by long-term metabolic syndrome. Therefore, future mechanistic priorities must shift toward validating the ALDH2-4-HNE axis in large animal models (e.g., porcine I/R models) presented with combined metabolic risk factors before accelerating clinical pilot trials.

## 7 Conclusion

In conclusion, ALDH2 is a critical endogenous cardioprotective enzyme in myocardial I/R injury. Its most direct function is the detoxification of reactive aldehydes such as 4-HNE and MDA, but the downstream impact is broader: preservation of mitochondrial structure and respiration, regulation of autophagy, attenuation of ferroptosis and apoptosis, limitation of necroptosis, and suppression of NETosis-related inflammatory injury. This integrated position makes ALDH2 a biologically plausible therapeutic target and an important modifier of individual susceptibility to reperfusion injury.

At the same time, the current literature has clear limitations. Most mechanistic evidence derives from animal or cellular models, the relative contribution of each pathway may differ by ischemic duration and reperfusion timing, and human data remain dominated by observational genotype or biomarker studies. The ALDH2*2 variant is highly relevant in East Asian populations, but the magnitude of benefit from targeted activation in different genotypes, sexes, comorbidity profiles, and treatment settings remains uncertain.

Future research should therefore move from proof-of-concept protection toward clinically testable strategies. Priorities include development of ALDH2-targeted agonists with suitable pharmacokinetic and safety profiles, prospective evaluation of ALDH2 polymorphisms for patient stratification, clarification of optimal timing around PCI or CABG, and rational combination with anti-ferroptotic, anti-NETosis, mitochondrial, or conditioning-based therapies. Only trials that integrate genotype, target-engagement biomarkers, and clinically meaningful remodeling or outcome endpoints will determine whether ALDH2 activation can become a practical therapeutic approach for myocardial I/R injury.
